# Influence of Telecommunication Modality, Internet Transmission Quality, and Accessories on Speech Perception in Cochlear Implant Users

**DOI:** 10.2196/jmir.6954

**Published:** 2017-04-24

**Authors:** Georgios Mantokoudis, Roger Koller, Jérémie Guignard, Marco Caversaccio, Martin Kompis, Pascal Senn

**Affiliations:** ^1^ lnselspital, Bern University Hospital Department of Otorhinolaryngology, Head and Neck Surgery University of Bern Bern Switzerland; ^2^ Department of Clinical Neurosciences, Service ORL and CCF University Hospital of Geneva Geneva Switzerland

**Keywords:** communication aids for disabled, telecommunications devices for the deaf, cochlear implants, speech discrimination tests, hearing loss, telephone

## Abstract

**Background:**

Telecommunication is limited or even impossible for more than one-thirds of all cochlear implant (CI) users.

**Objective:**

We sought therefore to study the impact of voice quality on speech perception with voice over Internet protocol (VoIP) under real and adverse network conditions.

**Methods:**

Telephone speech perception was assessed in 19 CI users (15-69 years, average 42 years), using the German HSM (Hochmair-Schulz-Moser) sentence test comparing Skype and conventional telephone (public switched telephone networks, PSTN) transmission using a personal computer (PC) and a digital enhanced cordless telecommunications (DECT) telephone dual device. Five different Internet transmission quality modes and four accessories (PC speakers, headphones, 3.5 mm jack audio cable, and induction loop) were compared. As a secondary outcome, the subjective perceived voice quality was assessed using the mean opinion score (MOS).

**Results:**

Speech telephone perception was significantly better (median 91.6%, *P*<.001) with Skype compared with PSTN (median 42.5%) under optimal conditions. Skype calls under adverse network conditions (data packet loss > 15%) were not superior to conventional telephony. In addition, there were no significant differences between the tested accessories (*P>*.05) using a PC. Coupling a Skype DECT phone device with an audio cable to the CI, however, resulted in higher speech perception (median 65%) and subjective MOS scores (3.2) than using PSTN (median 7.5%, *P*<.001).

**Conclusions:**

Skype calls significantly improve speech perception for CI users compared with conventional telephony under real network conditions. Listening accessories do not further improve listening experience. Current Skype DECT telephone devices do not fully offer technical advantages in voice quality.

## Introduction

A cochlear implant (CI) is an electronic device which allows an auditory stimulation in patients with severe or profound hearing loss [1,2]. It is the most successful neural prosthesis developed till date [1,3]. The implant consists of two parts, an external speech processor connected with a transmitting coil worn behind the ear and the implant itself placed under the skin behind the ear. The internal part of the implant receives the signals from the transmitting coil and sends electrical impulses to 12-22 electrodes, which are placed into the cochlea. The auditory nerve is hereby directly stimulated with high frequencies at the base of the cochlea and low frequencies at the apex [4]. CIs offer an improved hearing and quality of life [5]. Telecommunication, however, is limited or even impossible for more than one-third of CI users [6-10]. Assisting listening devices may improve speech perception performance, however, communication abilities still remain limited because of restricted frequency bandwidth (300-3400 Hz) and digital compression of voice data applied in conventional telephony (public switched telephone network, PSTN) [11,12]. Telephone speech perception might be additionally impaired by the coupling mode with a hearing aid or CI [13,14]. Ability to use a telephone is important for maintaining social contacts or in emergency situations. In addition, cognitive decline and dementia in older adults are often associated with hearing loss and reduced communication abilities [15-18]. Rehabilitation of hearing communication, however, improves cognitive function [19], quality of life and social participation, and any solution to improve telecommunication in CI patients should be pursued.

**Figure 1:**
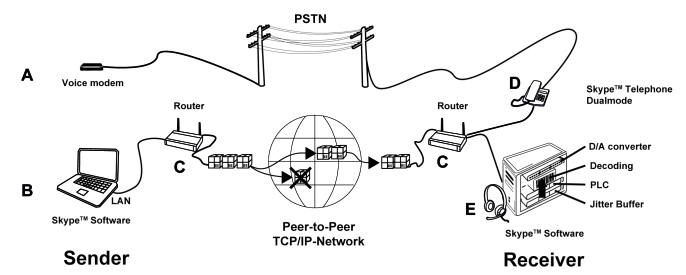
Test setup and VoIP. The standardized version of the HSM sentence test (for clinical testing) was sent from a CD player connected via an audio mixing console (XENYX 502 Behringer, Willich, Germany) to either a voice modem (A; Way2Call, Hi-Phone Desktop lite, Way2Call Communications Inc, Newmark, NJ, USA) or a laptop (B). Small voice data packets were sent from Skype PC software (B) through a router (C) over a transmission control protocol and Internet protocol network to the receiver. The receiver devices (D) or (E) branched to a router (C) collected all incoming data packets. The installed Skype PC software (E) or Skype App (D) was decoding the voice signal. A software controlled the number of lost data packets (0-20%), to induce different network scenarios. Alternatively, a conventional telephone line (PSTN connection) was used to transmit the audio signal from a voice modem (A) to a DECT telephone (D).

Recent laboratory studies showed advantages of Internet telephony (voice over Internet protocol, VOIP) with improved voice quality caused by a wider frequency bandwidth (200-8500Hz) than the conventional telephone [20,21]; however, all these studies were performed under laboratory conditions. There is currently no study showing this advantage under real network conditions. Software solutions such as Skype or Google Talk among others are supporting video telephony, which improves speech perception by adding visual cues [22]. In addition, VoIP software offers a wider range of transmitted frequencies (200Hz-12kHz, Figure 1) and should—in theory—offer a better voice quality and speech perception performance; however, limited Internet connection speed might deteriorate speech signals and voice quality [20,23,24]. The final transmitted voice quality depends on data transmission network capacities, delays, and the extension of data packet loss (PL) [25].

The aim of this study was to test telephone speech perception in CI users comparing the conventional telephone (PSTN) with VoIP (Skype) under real network conditions. Voice quality, coupling mode, accessories, and a Skype telephone digital enhanced cordless telecommunications (DECT) device were assessed to observe any improvement in distant communication.

## Methods

### Test Subjects

Nineteen adult CI users aged between 15 and 69 years (average 42 years) participated in the study. We included CI users with at least 6-month unilateral implant experience and either a Cochlear Nucleus Freedom or a CP810 Sound Processor fitted with a frequency allocation table reaching higher than 5 kHz. Each test subject had a minimal speech perception score of 50% for German monosyllabic words at 60 dB sound pressure level (SPL), 3 months after implantation. Table 1 shows patient characteristics of the included subjects. The study protocol was fully approved by the local institutional review board. All patients gave written informed consent.

**Table 1:** Clinical data of cochlear implant (CI) users.

ID	Age at measurement (years)	Gender	Cochlear implant Model	Speech processor	Age at implantation (years)	Years since implantation
1	51	Female	CI512	CP 810	50	1
2	24	Female	CI 512	CP 810	23	1
3	61	Male	CI 512	CP 810	59	2
4	40	Male	CI 24RE(CA)	Freedom	34	6
5	18	Male	CI 24RE(CA)	Freedom	12	6
6	15	Male	CI 24RE(CA)	Freedom	9	6
7	17	Male	CI 24RE(ST)	Freedom	12	5
8	63	Male	CI 512	CP 810	62	1
9	67	Female	Hybrid L24	Freedom	65	2
10	54	Female	CI 24RE(CA)	Freedom	48	6
11	25	Female	CI 24RE(CA)	Freedom	22	3
12	15	Female	CI 24RE(ST)	Freedom	12	3
13	31	Female	CI 24RE(CA)	Freedom	23	8
14	68	Male	Hybrid L24	Freedom	66	2
15	27	Male	CI 512	CP 810	26	1
16	55	Male	CI 512	CP 810	54	1
17	69	Male	CI 24R(CA)	CP 810	61	8
18	58	Female	CI 24(ST)	CP 810	48	10
19	40	Male	CI 422	CP 810	39	1

### Telephone Transmission Mode and Devices

We compared Internet telephony (Skype, local area network connection) against the conventional telephone (PSTN, landline connection) using two devices, a personal computer (PC; Latitude E6510, Dell, Round Rock, TX, USA) and a cordless DECT telephone device (Philips VoIP855, Royal Philips Electronics, Amsterdam, The Netherlands) which has dual transmission functions (Skype app and PSTN). In addition, we compared four accessories (PC speakers Z320; Logitech headphones, Behringer HPS 500; 3.5 mm jack audio cable, cochlear Ltd; and induction loop) and five different Internet transmission speeds (controlled with a connection emulator, Perfect Soft Research, Version 1.3.2 Brisbane, Australia). We used Internet connections with random data PL in 5% steps ranging from 0% PL (perfect), 5% PL (mild), 10% PL (medium), 15% (severe) to 20% PL (very severe). Figure 1 shows the test setup with the two transmission modes (PSTN versus Skype) connected with two devices (PC, Figure 1 E and cordless DECT telephone, Figure 1 D).

The frequency response was measured for all devices using an audio analyzer (UPV, Rhode & Schwarz, Munich, Germany) and a head and torso simulator (KEMAR Manikin Type 45BA, Brüel & Kjaer, Naerum, Denmark). For acoustic measurements, the Manikin’s ear simulator (Type 4158) was used to simulate the situation of a telephone held on the ear. The ear simulator is composed of a silicon external ear (or pinna) coupled to an ear canal terminated by a half-inch condenser microphone and pre-amplifier. A sweep of 50 logarithmically spread, pure sinus tones was generated and the output was filtered with a 1% bandwidth filter locked on the stimulus frequency. The root mean square amplitude of the output was calculated to create a frequency-domain graph. Objective voice quality was tested using the audio analyzer’s built-in algorithm for Perceptual Evaluation of Speech Quality (PESQ) based on the guidelines of the ITU (International Telecommunication Union) in accordance with the ITU-T P.862 protocol. Test subjects rated the subjective perceived voice quality from 1-5 using the mean opinion score (MOS; Table 2, according to specifications ITU-T Rec. P.862.1 and P.862.2) [26].

**Table 2:** Mean Opinion Score (MOS) for subjective voice quality assessment.

Score	Quality	Listening effort scale
5	Excellent	No effort required
4	Good	No appreciable effort required
3	Fair	Moderate effort required
2	Poor	Considerable effort required
1	Bad	No meaning understood with reasonable effort

### Speech Perception Test Protocol

The standardized German “HSM” sentence test [27] was used for open set monosyllable speech perception testing in noise (60 dB SPL) at a constant signal sound level of 70 dB SPL (free field at 1-m distance). The HSM test consists of 30 lists with 20 short sentences containing 106 monosyllable words. The order of the sentence was changed at random to avoid learning effects. The subject had to repeat the presented sentences, and received 1 point for each correctly reproduced word. The percent of speech perception from a total of 106 words was calculated for each condition (one list). Test subjects were tested monaurally in a sound treated room (Type 402A, Industrial Acoustics Company, Niederkrüchten, Germany), with an average reverberation time of 0.10 s (125-10000 Hz). The contralateral ear canal was closed by an earplug (USA EARlink 3C, EAR Corporation, Indianapolis) if there was a residual hearing. Other hearing aids or a second CI had to be turned off. Patients kept the everyday settings of their speech processor. The cordless DECT telephone (Figure 1 D) was coupled to the implant either with an audio cable (Personal Audio Cable, Cochlear Limited, Sydney, Australia) branched to the 3.5-mm jack socket of the handset or by holding it directly to the ear. We chose this experimental setting with speech signal presented in noise not only to simulate an everyday listening situation but also to avoid ceiling effects.

### Statistics

Robust nonparametric analyses were performed to assess the potentially non-normally distributed speech perception scores from this small study population. A two-tailed Wilcoxon matched pairs signed-rank test was used to compare Skype with PSTN. For the ideal condition with no Internet data PL (condition 0% PL), a one-tailed test was applied because of the expected superiority of Skype under this condition [21,23]. A *P* value<.05 was considered significant after applying a Bonferroni correction for multiple testing.

## Results

### Telephone Transmission Mode and Voice Quality

**Figure 2:**
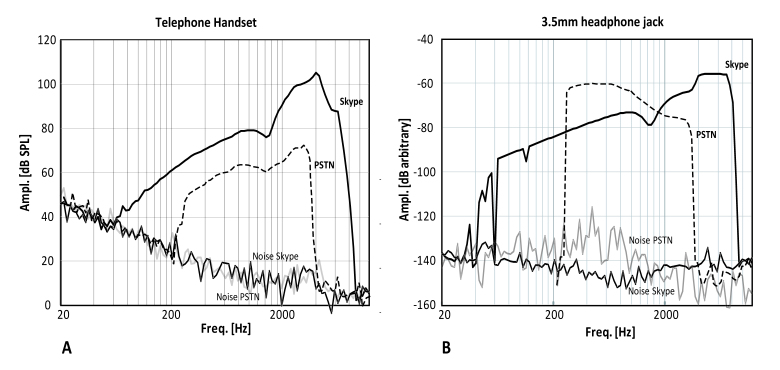
Frequency response for Skype and telephone. It shows the frequency response of the conventional telephone (PSTN) and Skype (LAN connection). The transmitted audio signal was tested using an audio analyzer and a head and torso simulator. The x-axis shows the logarithmic frequency scale, and the y-axis, the recorded sound pressure level or electrical output from either the handset loudspeaker (panel A) or the handset 3.5-mm headphone jack (panel B) of the telephone.

The differences of the transmitted frequencies of PSTN versus Skype (LAN connection) are shown in Figure 2. There was a better frequency response of the audio signal derived from the headphone jack compared with the telephone handset (Figure 2). A broader frequency range (50–5000 Hz) was transmitted via Skype than PSTN (200-3000 Hz) independently of whether the built-in loudspeaker or the headphone jack was used.

**Figure 3:**
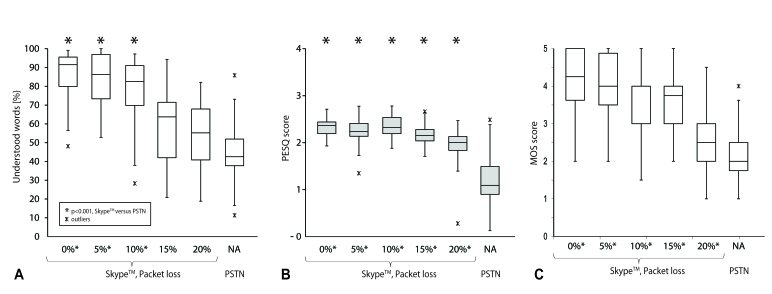
Speech perception and transmission mode. Box plots demonstrating lower quartile, median, and upper quartile, and whiskers representing 1.5 times the IQR (X=outliers): Free-field speech perception performance (correctly repeated words in percentage) from 19 CI users at 5 different Skype transmissions with 0-20% data PL and one landline connection (PSTN). Stars indicate a statistically significant difference between a Skype and a PSTN transmission. PESQ scores measurements (panel B) (assessed with an audio analyzer) indicate the objective measured voice quality (y-axis) for the different test conditions (Skype connection with data PL from 0% to 20% and PSTN). Panel C shows the subjective perceived voice quality under the same conditions.

Speech perception with an optimal Skype connection (0% PL; median 91.6%, n=18, range 48.1-99.1%; interquartile range, IQR, 15.6) which was significantly better (*P*<.001) than the telephone speech perception (PSTN median 42.5%, n=18, range 11.3- 85.8%, IQR 15.6); however, there was statistically no advantage in speech understanding using Skype at lower Internet quality connections (PL 15% and 20%; Figure 3). Quality measurements of the transmitted sound (PESQ measurement) showed that all Skype quality connections (PL 0- 20%) offered a significantly better voice quality (*P*<.001) compared to PSTN (Figure 3). Skype voice quality was maintained even with a medium quality Internet connection (PL 5% and 10%; Figure 3)

### VoIP Accessories for PC and CI

**Figure 4:**
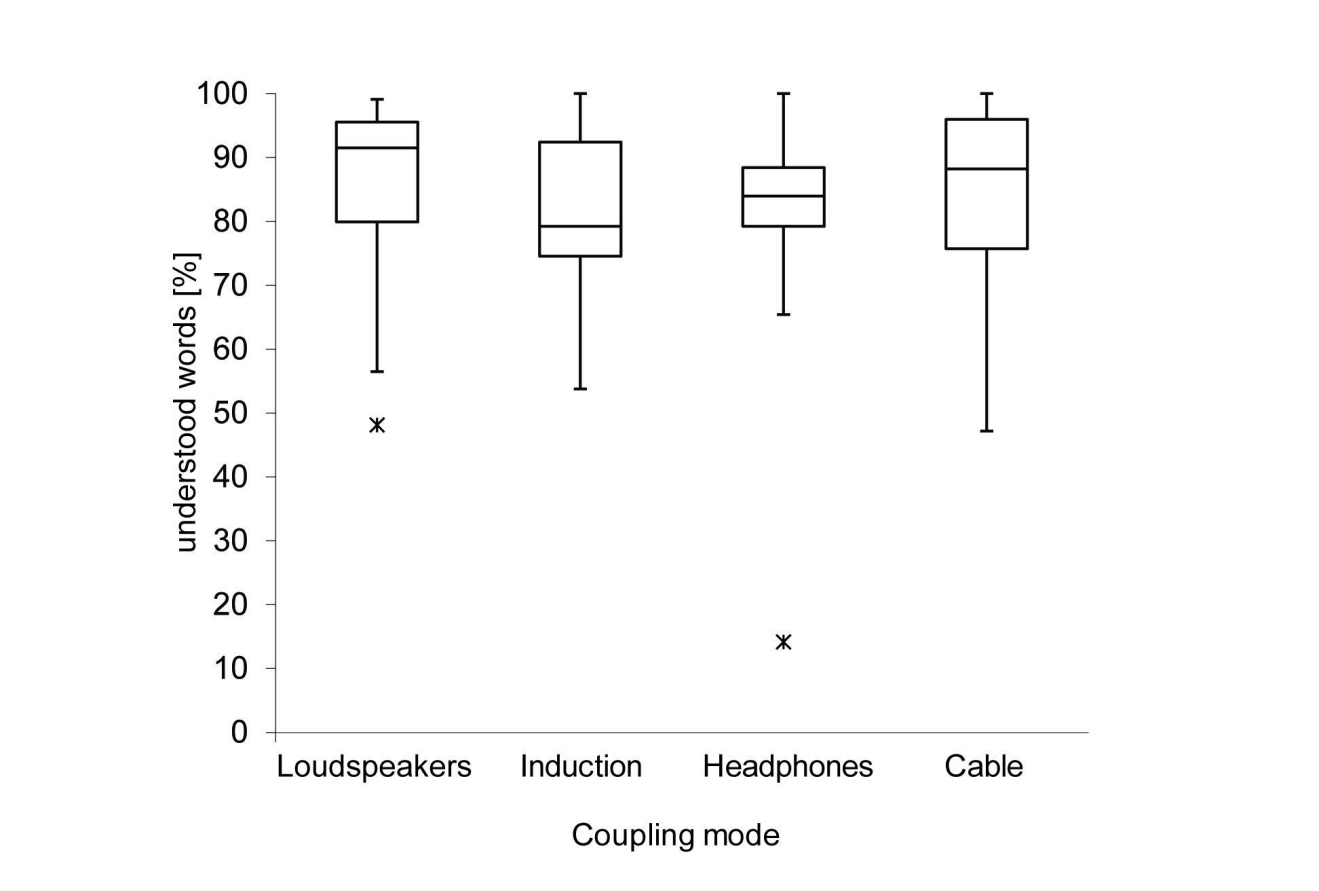
Speech perception scores using telephone accessories. Four different accessories have been compared in terms of speech perception under optimal Internet connections (0% data PL): an induction loop, headphones, an audio cable, and active loudspeakers connected to a Skype PC have been tested. Box plots are indicating the median percentage of word recognition. There was no significant difference across the tested accessories.

Free-field speech perception with Skype (PC version) using an optimal Internet connection (0% PL) was 91.5 % (median, n=18, range 48.1-99.1 %, IQR 15.6). Speech perception with a connected induction loop was 79.3% (median, n=15, range 53.8-100%, IQR 17.92), with headphones 83.9 % (median, n=18, range 14.2-100 %, IQR 9.215), and with coupled CI cables 88.2 % (median, n=18, range 47.1-100 %, IQR 20.27). There was no significant difference (*P>*.05) between the tested accessories (Figure 4).

### Skype DECT Phone Device and Coupling Mode

**Figure 5:**
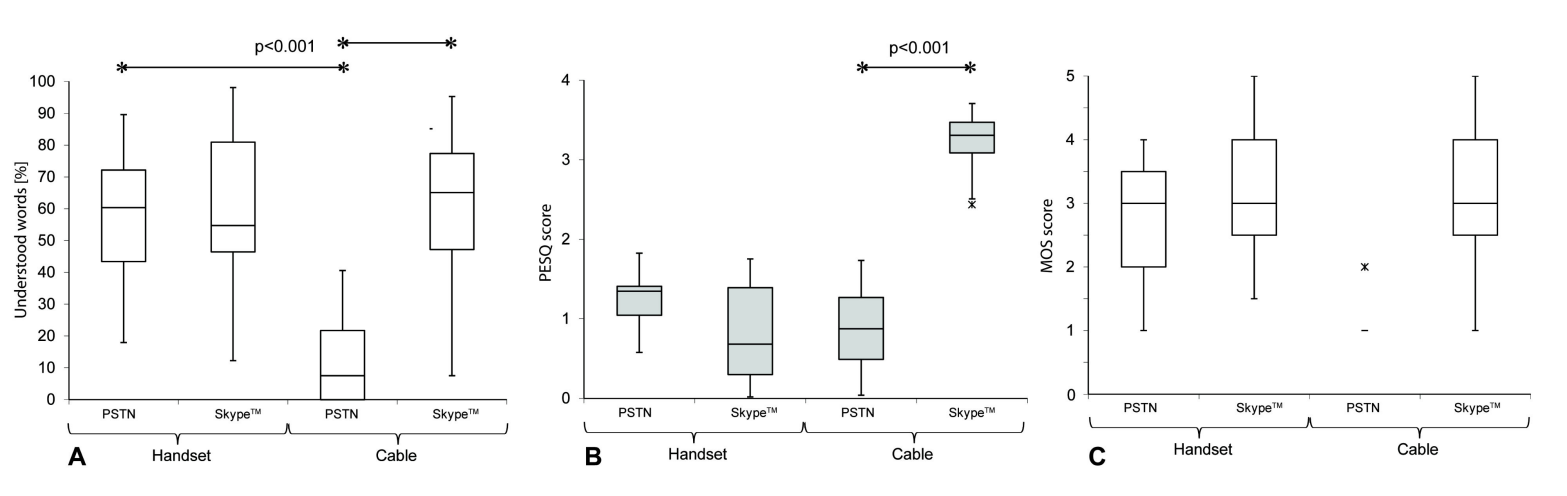
Speech perception and coupling mode. It shows speech perception scores using a DECT phone with dual transmission modes (either PSTN or Skype). The handset was either coupled to the CI microphone above the ear (handset coupling mode) or connected directly over the 3.5-mm headphone jack to the CI (cable coupling). Stars indicate significant differences. The objective voice quality (y-axis) was measured on a head and torso simulator for the same conditions (panel B). The subjective perceived voice quality (MOS) was rated by the participants for each condition (panel C).

Figure 5 shows the speech perception performance with a DECT telephone coupled to the CI either with a cable or the handset. Median speech perception using a CI audio cable was 7.5% for PSTN (n=17, range 0-40.6%, IQR 21.7) compared with speech understanding of 65.1% with the Skype app (median, n=17, range 47.17-95.3%, IQR 30.2) installed on the DECT telephone. Speech understanding with Skype was significantly superior if the DECT telephone was directly coupled to the CI by cable (*P*<.001). There was, however, no significant advantage seen for Skype if the handset was held directly near the microphone above the pinna (Figure 5).

The voice quality measurements (PESQ) on the headphone jack socket of a DECT phone (cable connection) showed significantly higher scores for the installed Skype app (median 3.08, n=19, range 2.43- 3.71, IQR 0.285) compared with the PSTN connection (median 0.73, n=19, range 0.33-1.73, IQR 0.94, *P*<.001) on the same device (Figure 5). The built-in telephone loudspeakers of the handset, however, did not show any significant voice quality differences between PSTN and the Skype app (Figure 5).

### Mean Opinion Score (MOS)

The subjective perceived voice quality, which describes the level of effort required for understanding, was best with an optimal Skype connection (MOS 4.1 [SD 0.9] at PL 0%; Table 3; Figure 3). The audio cable was rated as the best accessory requiring the least effort for understanding speech (MOS 4.2, SD 0.7), while the neck loop was found to offer the most unpleasant sound (MOS 3.5, SD 1.2). Figures 3 and 5 show a comparison between psychoacoustic (panels A), technical (panels B), and subjective (panels C) performance.

**Table 3:** Mean opinion scores (MOS) for each modality

Modality	Method	Mean opinion scores (MOS)^a^
**VoIP vs PSTN**		
	Skype connection PL 0%	4.1
	Skype connection PL 5%	4
	Skype connection PL 10%	3.6
	Skype connection PL 15%	3.6
	Skype connection PL 20%	2.6
	Telephone connection (PSTN)	2.1
**Accessories**		
	PC-Speakers	4.1
	Headphones	3.9
	Neck Induction Loop	3.5
	Audio cable	4.2
**Cordless DECT telephones**		
	PSTN Handset coupling	2.9
	PSTN Audio cable	1.2
	Skype Handset coupling	3.2
	Skype Audio cable	3.2

^a^Subjective perceived voice quality was assessed using a 5-point unipolar rating scale (MOS, Table 2), ranging from 5 points indicating an excellent voice quality to 1 point indicating a bad voice quality.

## Discussion

### Principal Findings

Speech perception by CI patients using Skype with active loudspeakers was superior to the conventional telephone under perfect or medium network voice transmissions. There was no advantage of Internet telephony for transmissions with severe or very severe data PL (>10% PL). Accessories such as a neck loop with wireless telecoil, an audio cable directly connected to the CI or headphones, did not further improve speech perception; however, the CI users subjectively perceived the voice quality (MOS) as superior, when using direct cable input. A dedicated Skype DECT telephone did not offer the full advantage of the superior voice quality provided by the Skype app because of loudspeaker quality limitations.

### Impact of Internet Connection Quality

A proof of concept was given by a previous experimental study showing a superior voice quality of Internet telephony resulting in better speech understanding, provided that the Internet connection quality was ideal [21]. This phenomenon was also shown in normal hearing subjects and was not related to any speech coding strategy of a CI [21].

Sound quality might be affected by low bit-rate coding, data PL, background noise, silence suppression, or by network filtering leading to sound delay, jitter, noise, and speech level changes. These parameters might not equally influence speech perception performance by CI users. The PESQ score, however, uses objective parameters to model psychoacoustic and cognitive perception of speech. Such a model was calibrated to predict MOS scores and to improve correlation between PESQ and MOS scores [26].

Live Skype calls (desktop version) transmitted via a deteriorated Internet connection (5-10% PL) still generated a better voice quality on the receiver side compared with a PSTN connection, but not for transmission modes with severe PL (15% and 20%). These findings are in line with speech perception test results performed under simulated laboratory conditions [20].

The main reason for the superiority of VoIP is likely due to technical reasons. The frequency coverage with VoIP is higher than that of conventional telephony (0.1-8 kHz vs 0.3-3.4 kHz; Figure 2) resulting in better audio quality (see PESQ measurements). Since Skype transmits higher frequencies, it is likely to convey more of the speech-relevant signal content such as consonants. In addition, telecommunication companies digitalize the analogue voice signal using low bit-rate coding (8 kHz sampling rate, G.711 codec, ITU recommendations, G-series) and maximum bit rates of 33.6 kbits/s compared with Skype, which uses a sampling rate of 16 kHz and variable bit rates up to 40 kbits/s [28].

No improvement in speech perception, however, was measured for the CI users when PL was >10% (Figure 3). This was unexpected after analyzing objective voice quality measurements (PESQ), which proved a better audio quality of Skype, regardless of the Internet connection quality (Figure 3). All tests were performed at a constant background noise, making these tests more sensitive to speech signal deterioration, which might have an impact on speech perception performance.

### Telephone Listening Accessories

Accessories may improve telephone listening experience in background noise [29]. We measured no significant speech discrimination differences whether accessories were used or not. Possible ceiling effects might have occurred, since median speech perception performance was around 90% regardless of the accessory used. Test conditions with a lower signal-to-noise ratio (SNR < 10 dB) might have yielded different performance results since assisting listening accessories protect from unwanted noise by either shielding physically (around-the-ear/circumaural headphones) or by routing the signal directly to the device (cable, induction, FM system). Loudspeakers, however, do not offer an improved SNR, which is important for speech discrimination in CI users.

We tested only monaurally to simulate an everyday telephone use, and this might have affected the general performance with accessories. Studies show significant advantages in speech perception with binaural hearing (particularly in environmental noise) [30] or even in bimodal hearing [31]. Assistive listening devices enable users to transmit sound on both ears simultaneously.

Our data suggest that the choice of accessories should be made according to personal preference. Low quality built-in loudspeakers of the Skype DECT device resulted in lower voice quality and consequently in a lower speech perception performance. The Skype DECT device offered a better speech perception if the headphone jack was used. Most subjects also reported the best subjective listening experience with the audio cable connecting the headphone jack with the CI, although a cable connection limits the range of use. This might change with new wireless streaming possibilities [29,32,33], which have not been tested yet with VoIP applications.

### Strength and Limitations

This study analyzed telephone speech perception using real-time settings and measurements, which give a better estimate than the previous laboratory tests.

The patients kept the everyday settings of their speech processor, however, another way to improve the telephone listening experience is the fine structure preservation [6] or the application of a special telephone fitting mode [34] by reducing the current level for electrodes stimulating outside the transmitted frequency range.

Although all individuals were blinded regarding the tested condition, performance bias might still be possible, since individuals were aware of the used accessories or might have perceived the presented voice quality. The small sample size might lead to an under- or overestimation of study results. In addition, this is a self-controlled study without any other control group.

We tested one single VoIP software and selected accessories. These results are therefore not generalizable for all available VoIP programs or other Internet transmissions modes.

Further research is mandatory to test voice quality and speech perception performance through wireless and mobile Internet connections. New technologies such as wireless audio streaming [35] may further improve listening experience and performance. New generations of Bluetooth technology with low battery consumption and direct connection to the implant might replace any assisting telephone accessory in the future while preserving voice quality.

### Clinical Implications

Internet telephony improves speech perception performance even under real and adverse network conditions. CI users who are not able to have a meaningful telephone conversation could improve their telephone listening experience by using Skype or any other broadband Internet telephony service. This might have a direct impact on social integration, general health, life expectancy [16], and cognitive function in the elderly [18,36,37]. Restrictions in interactions and activities because of hearing loss might result in reduction of the overall health status and thus increase morbidity and mortality [16].

Skype and other VoIP software are freely available and can be used with any computer, microphone and speaker system. Additional assisting listening devices and telephone accessories might help for binaural hearing or hearing in noise.

### Conclusions

Broadband VoIP software such as Skype can significantly improve telecommunication experience for CI users even with low quality Internet connections. Listening accessories such as headphones, audio cables, or an induction loop were equivalent in terms of speech perception performance. Microphone and speaker quality of Skype telephone DECT devices do not fully exploit benefits of Skype apps which provide an enhanced broadband audio and voice quality.

## Abbreviations

CI: cochlear implant
DECT: digital enhanced cordless telecommunications
IQR: interquartile range
ITU: International Telecommunication Union
MOS: mean opinion score
PESQ: perceptual evaluation of speech quality
PL: packet loss
PSTN: public switched telephone networks
SNR: signal-to-noise ratio
SPL: sound pressure level
VoIP: voice over Internet protocol

## References

[1] Géléoc GS, Holt JR (2014). Sound strategies for hearing restoration. Science.

[2] Basura GJ, Eapen R, Buchman CA (2009). Bilateral cochlear implantation: current concepts, indications, and results. Laryngoscope.

[3] Brand Y, Senn P, Kompis M, Dillier N, Allum JH (2014). Cochlear implantation in children and adults in Switzerland. Swiss Med Wkly.

[4] Hochmair I, Hochmair E, Nopp P, Waller M, Jolly C (2015). Deep electrode insertion and sound coding in cochlear implants. Hear Res.

[5] Gaylor J, Raman G, Chung M, Lee J, Rao M, Lau J, Poe DS (2013). Cochlear implantation in adults: a systematic review and meta-analysis. JAMA Otolaryngol Head Neck Surg.

[6] Galindo J, Lassaletta L, Mora RP, Castro A, Bastarrica M, Gavilán J (2013). Fine structure processing improves telephone speech perception in cochlear implant users. Eur Arch Otorhinolaryngol.

[7] Holmes AE, Frank T (1984). Telephone listening ability for hearing-impaired individuals. Ear Hear.

[8] Kepler LJ, Terry M, Sweetman RH (1992). Telephone usage in the hearing-impaired population. Ear Hear.

[9] Adams J, Hasenstab MS, Pippin GW, Sismanis A (2004). Telephone use and understanding in patients with cochlear implants. Ear Nose Throat J.

[10] Wu C, Liu T, Wang N, Chao W (2013). Speech perception and communication ability over the telephone by Mandarin-speaking children with cochlear implants. Int J Pediatr Otorhinolaryngol.

[11] Liu C, Fu QJ, Narayanan SS (2009). Effect of bandwidth extension to telephone speech recognition in cochlear implant users. J Acoust Soc Am.

[12] Milchard AJ, Cullington HE (2004). An investigation into the effect of limiting the frequency bandwidth of speech on speech recognition in adult cochlear implant users. Int J Audiol.

[13] Ito J, Nakatake M, Fujita S (1999). Hearing ability by telephone of patients with cochlear implants. Otolaryngol Head Neck Surg.

[14] Cray JW, Allen RL, Stuart A, Hudson S, Layman E, Givens GD (2004). An investigation of telephone use among cochlear implant recipients. Am J Audiol.

[15] Dalton DS, Cruickshanks KJ, Klein BE, Klein R, Wiley TL, Nondahl DM (2003). The impact of hearing loss on quality of life in older adults. Gerontologist.

[16] Barnett S, Franks P (1999). Deafness and mortality: analyses of linked data from the National Health Interview Survey and National Death Index. Public Health Rep.

[17] Cacciatore F, Napoli C, Abete P, Marciano E, Triassi M, Rengo F (1999). Quality of life determinants and hearing function in an elderly population: osservatorio geriatrico campano study group. Gerontology.

[18] Gates GA, Cobb JL, Linn RT, Rees T, Wolf PA, D'Agostino RB (1996). Central auditory dysfunction, cognitive dysfunction, and dementia in older people. Arch Otolaryngol Head Neck Surg.

[19] Mosnier I, Bebear J, Marx M, Fraysse B, Truy E, Lina-Granade G, Mondain M, Sterkers-Artières F, Bordure P, Robier A, Godey B, Meyer B, Frachet B, Poncet-Wallet C, Bouccara D, Sterkers O (2015). Improvement of cognitive function after cochlear implantation in elderly patients. JAMA Otolaryngol Head Neck Surg.

[20] Mantokoudis G, Dubach P, Pfiffner F, Kompis M, Caversaccio M, Senn P (2012). Speech perception benefits of internet versus conventional telephony for hearing-impaired individuals. J Med Internet Res.

[21] Mantokoudis G, Kompis M, Dubach P, Caversaccio M, Senn P (2010). How internet telephony could improve communication for hearing-impaired individuals. Otol Neurotol.

[22] Mantokoudis G, Dähler C, Dubach P, Kompis M, Caversaccio MD, Senn P (2013). Internet video telephony allows speech reading by deaf individuals and improves speech perception by cochlear implant users. PLoS One.

[23] Trond U, Stafnsnes F (2006). VoIP speech quality - better than PSTN?. Telektronikk.

[24] Sun LF, Wade G, Lines BM, Ifeachor EC (2001). Impact of packet loss location on perceived speech quality.

[25] Ding L, Goubran RA (2003). Assessment of effects of packet loss on speech quality in VoIP.

[26] Rix AW, Beerends JG, Hollier MP, Hekstra AP (2001). Perceptual evaluation of speech quality (PESQ) - a new method for speech quality assessment of telephone networks and codecs.

[27] Hochmair-Desoyer I, Schulz E, Moser L, Schmidt M (1997). The HSM sentence test as a tool for evaluating the speech understanding in noise of cochlear implant users. Am J Otol.

[28] Vos K, Jensen S, Soerensen K IETF.

[29] Kim JS, Kim CH (2014). A review of assistive listening device and digital wireless technology for hearing instruments. Korean J Audiol.

[30] Litovsky RY, Goupell MJ, Godar S, Grieco-Calub T, Jones GL, Garadat SN, Agrawal S, Kan A, Todd A, Hess C, Misurelli S (2012). Studies on bilateral cochlear implants at the University of Wisconsin's binaural hearing and speech laboratory. J Am Acad Audiol.

[31] Iwaki T, Matsushiro N, Mah S, Sato T, Yasuoka E, Yamamoto K, Kubo T (2004). Comparison of speech perception between monaural and binaural hearing in cochlear implant patients. Acta Otolaryngol.

[32] Wolfe J, Morais M, Schafer E (2016). Speech recognition of bimodal cochlear implant recipients using a wireless audio streaming accessory for the telephone. Otol Neurotol.

[33] Qian H, Loizou PC, Dorman MF (2003). A phone-assistive device based on Bluetooth technology for cochlear implant users. IEEE Trans Neural Syst Rehabil Eng.

[34] Di NW, Anzivino R, Gambini G, De CE, Paludetti G (2014). Improvement of telephone communication in elderly cochlear implant patients. Audiol Neurootol.

[35] Wolfe J, Morais DM, Schafer E, Cire G, Menapace C, O'Neill L (2016). Evaluation of a wireless audio streaming accessory to improve mobile telephone performance of cochlear implant users. Int J Audiol.

[36] Lin FR, Yaffe K, Xia J, Xue Q, Harris TB, Purchase-Helzner E, Satterfield S, Ayonayon HN, Ferrucci L, Simonsick EM, Health ABC Study Group (2013). Hearing loss and cognitive decline in older adults. JAMA Intern Med.

[37] Wayne R, Johnsrude IS (2015). A review of causal mechanisms underlying the link between age-related hearing loss and cognitive decline. Ageing Res Rev.

